# Association between genetic proxies for neuroticism and labour market outcomes

**DOI:** 10.1192/j.eurpsy.2025.297

**Published:** 2025-08-26

**Authors:** A. Hazak, J. Liuhanen, K. Kantojärvi, S. Sulkava, T. Jääskeläinen, V. Salomaa, S. Koskinen, M. Perola, T. Paunio

**Affiliations:** 1 Department of Public Health and Welfare, Finnish Institute for Health and Welfare; 2 SleepWell Research Program, University of Helsinki, Helsinki; 3 Department of Finance, Aalto University, Helsinki area, Finland; 4 Department of Economics and Finance, Tallinn University of Technology, Tallinn, Estonia; 5 Department of Psychiatry / SleepWell Research Program, University of Helsinki; 6 Department of Psychiatry; 7 Department of Psychiatry / Department of Clinical Genetics, Helsinki University Central Hospital; 8 Research Program for Clinical and Molecular Metabolism, University of Helsinki, Helsinki, Finland

## Abstract

**Introduction:**

Neuroticism, defined by a tendency to experience negative emotions such as anxiety and irritability, has significant public health implications, affecting both mental and physical health. It is also associated with poor career outcomes, including lower job satisfaction and higher rates of occupational failure, highlighting its importance in understanding labour market disparities. Despite advances in genetics, including insights into genetic background for neuroticism through large genome-wide association studies, the relationship between genetic predisposition to neuroticism and labour market performance remains underexplored.

**Objectives:**

This study examines the association between polygenic score for neuroticism (Nagel et al. *Nat Genet* 2018; 50 920-927) and individual labour market performance over 25 years in Finland. We aim to uncover how genetic predisposition to neuroticism relates to socio-economic outcomes, contributing to a broader understanding of its public health and economic implications.

**Methods:**

Our analysis draws on pooled data from the Finnish Finrisk (1992-2012) and FinHealth (2017) studies, comprising a representative sample of adults aged 25-64 (N=20,121). We integrated genetic, survey and socio-economic registry data. Using probit and semi-structural regression models, we assessed labour market outcomes, with neuroticism polygenic scores as the primary explanatory variable. Controls for socio-demographic factors and genetic principal components were included.

**Results:**

Individuals with higher polygenic scores for neuroticism were found to have lower income, partly through a direct and partly through a mediated association. Higher polygenic risk for neuroticism was linked to a decreased likelihood of completing higher education. Additionally, our study provides novel insights into how elevated polygenic risk for neuroticism was linked to labour market status.

**Conclusions:**

Our findings demonstrate that genetic predisposition for neuroticism is not only associated with educational setbacks but also translates into labour market disadvantages. While the effect sizes are modest, our results suggest that compensatory measures and support may help mitigate the career disadvantages faced by individuals with a higher genetic risk for neuroticism.

**Disclosure of Interest:**

A. Hazak: None Declared, J. Liuhanen: None Declared, K. Kantojärvi: None Declared, S. Sulkava: None Declared, T. Jääskeläinen: None Declared, V. Salomaa: None Declared, S. Koskinen: None Declared, M. Perola: None Declared, T. Paunio Consultant of: Idorsia Pharmaceuticals and Biogen (unrelated to the present work).

